# Epidemic of fractures during a period of snow and ice: has anything changed 33 years on?

**DOI:** 10.1136/bmjopen-2015-010582

**Published:** 2016-09-14

**Authors:** Waheeb Al-Azzani, Danial Adam Maliq Mak, Paul Hodgson, Rhodri Williams

**Affiliations:** 1 University Hospital of Wales, Cardiff, UK; 2 Cardiff University, Cardiff, UK; 3 University Hospital Llandough, Penarth, UK; 4 Hywel Dda University Health Board, Llanelli, UK

**Keywords:** snow, ice, fracture, incidence, weather

## Abstract

**Objectives:**

We reproduced a frequently cited study that was published in the *British Medical Journal* (*BMJ*) in 1981 assessing the extent of ‘snow-and-ice’ fractures during the winter period.

**Setting:**

This study aims to provide an insight into how things have changed within the same emergency department (ED) by comparing the findings of the *BMJ* paper published 33 years ago with the present date.

**Participants:**

As per the original study, all patients presenting to the ED with a radiological evidence of fracture during three different 4-day periods were included. The three 4-day periods included 4 days of snow-and-ice conditions and two control 4-day periods when snow and ice was not present; the first was 4 days within the same year, with a similar amount of sunshine hours, and the second was 4 days 1 calendar year later.

**Primary and secondary outcome measures:**

To identify the frequency, distribution and pattern of fractures sustained in snow-and-ice conditions compared to control conditions as well as comparisons with the index study 33 years ago.

**Results:**

A total of 293 patients with fractures were identified. Overall, there was a 2.20 (CI 1.7 to 3.0, p <0.01) increase in risk of fracture during snow-and-ice periods compared to control conditions. There was an increase (p <0.01) of fractures of the arm, forearm and wrist (RR 3.2 (CI 1.4 to 7.6) and 2.9 (CI 1.5 to 5.4) respectively).

**Conclusions:**

While the relative risk was not of the magnitude 33 years ago, the overall number of patients presenting with a fracture during snow-and-ice conditions remains more than double compared to control conditions. This highlights the need for improved understanding of the impact of increased fracture burden on hospitals and more effective preventative measures.

**Strengths and limitations of this study**

- The increase in incidence of fracture during snow-and-ice condition is predictable and preventable. This issue was highlighted 33 years ago in a study from our institution.
- We replicated the original study to see whether or not there have been any changes.
- A significant increase in fractures in relation to the presence of snow and ice continues to be identified.
- By recreating the methodology of the original paper, we inherited its limitations.
- The mechanism of injury and the causality relationship between variation in weather and risk of fracture were not explored.

## Introduction

Winter pressures on hospitals are well established. This includes the trauma burden of fractures associated with snow and ice.^1–5^ Incidence of fracture injuries during such conditions is predictable and potentially preventable. Thirty-three years ago, Ralis published his paper in the *British Medical Journal* (*BMJ*) on fracture incidence in snow and ice looking at data from our University Teaching Hospital emergency department (ED).^3^ At the time, our ED had a turnover of ∼95 000 patients per year.^3^ Today, it remains one of the busiest in the UK treating over 125 000 patients per year.^6^

We reproduced Ralis’ frequently cited study, in the same ED, published in the *BMJ* 33 years ago, to assess the extent of ‘snow-and-ice’ fractures during the winter and to see how things may have changed.

## Methods

We adopted the same methodology of the original study. A period of 4 days of snow and ice was compared with two control periods. Snow-and-ice days were defined, as per the original study, as having more than 70% of snow-and-ice coverage in the Cardiff area. This information was obtained via the Freedom of Information Act (2000) requests from the Meteorological Office and Cardiff County Council. These were Friday to Monday 18 to 21 January 2013. These also represented the coldest consecutive 4 days in 2013 with an average minimum temperature of −3°C. The first control period represented four matching days of the week with comparable hours of daylight to the snow-and-ice days (Friday 1 to Monday 4 February 2013). The second control period mirrored the same week days as the snow-and-ice days 1 year later (Friday 17 to Monday 20 January 2014), however, with milder conditions without snow and ice.

The radiological reports of every patient who underwent any form of imaging in our ED on the snow-and-ice and control days were identified and reviewed by the author (WA). The initial identification method of all patients present to the ED was via the Patient Management System. The subsequently reviewed radiology reports and images were available on the Patient Archive and Communication System. The imaging of patients identified as having a fracture were then viewed by the author (WA) to validate the presence of a fracture.

All patients with radiological evidence of fracture were included in this study. Patients were grouped and analysed according to their sex, age and anatomical site of injury. As per the original study, age groups were as follows: children (0–15 years), young adults (16–30 years), adults (31–60 years) and people aged 61 years and over. In addition, anatomical sites of injury were categorised as the head, arm, forearm and wrist, hand, spine and chest, hip, leg, and ankle and foot.

The primary outcome measures of this study were as follows:

1. To identify the current frequency, distribution and pattern of fractures sustained in snow-and-ice conditions compared to control conditions,
2. To compare frequency, distribution and pattern of fractures sustained in snow-and-ice conditions nowadays to those of 33 years ago.

Relative risks were calculated as the ratio of the probability of presenting with a fracture to ED in those exposed to snow-and-ice conditions to the probability of a fracture occurring in non-snow-and-ice conditions. IBM SPSS Statistics Software (V.21.0, Armonk, New York, USA: IBM Corp) was used to calculate CIs and p values.

## Results

**Figure 1:**
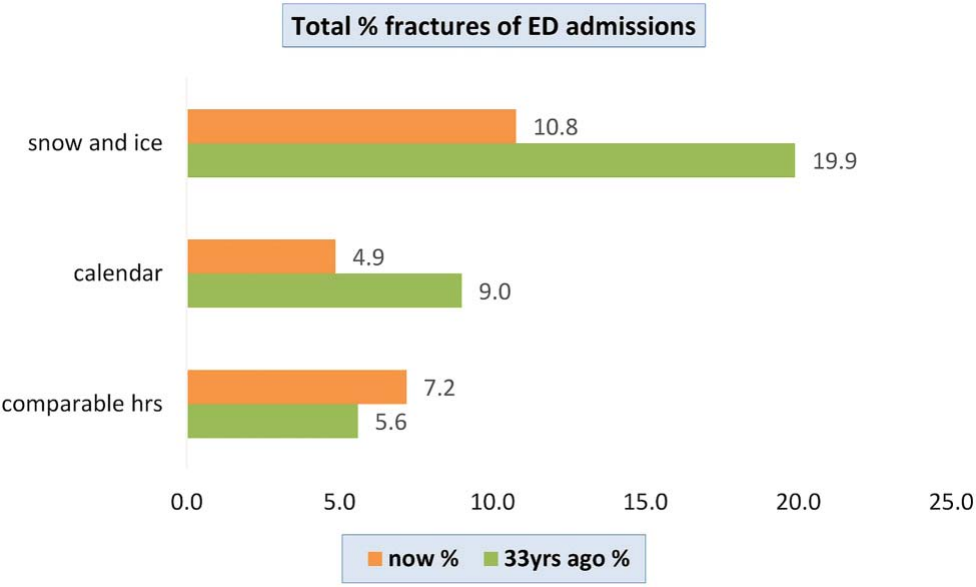
Proportion of fractures presented to ED on each day group.

In total, 1151 patients presented to ED during the 4 snow-and-ice days, 1474 during the 4 control days with comparable hours of sunlight and 1293 during the 4 control days 1 year later. The number of fractures sustained on snow-and-ice days, days with comparable sunlight hours and control days 1 year later were 124 (10.8%), 106 (7.2%) and 63 (4.9%), respectively (figure 1). This compares to 19.9%, 5.6% and 9% sustaining fractures during snow and ice, days with comparable sunlight hours and control days, respectively, 33 years ago. This equates to a 2.20 (CI 1.7 to 3.0, p<0.01) increase in risk of fracture during snow-and-ice periods compared to 2.85 during the same period 33 years ago. Figure 1 shows the proportion of fractures presented to ED for each group.

**Figure 2:**
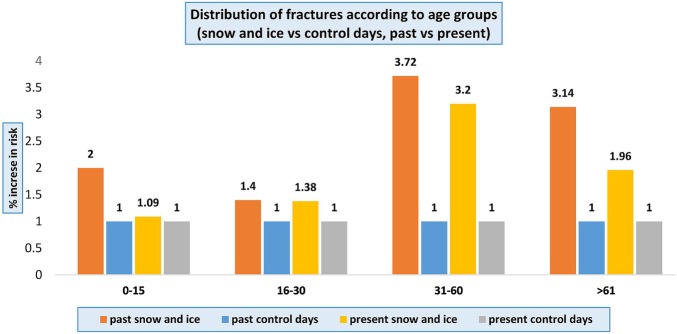
Percentage increase in risk of fractures according to age during snow-and-ice and control days comparing now to 33 years ago.

**Figure 3:**
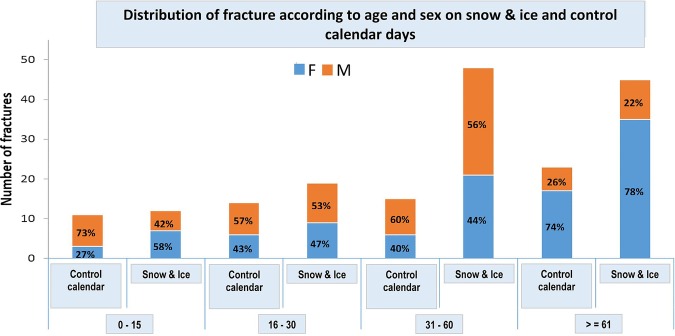
Percentage increase in frequency of fractures according to sex during snow-and-ice and control days.

Similar to findings 33 years ago, adults aged 31 years and above presented with fractures up to three times more frequently compared to the younger groups during snow-and-ice days. In the group aged over 61 years, women were 3.5 times more common to present with fracture compared to men. Figures 2 and 3 show the percentage increase in risk of fractures according to age and sex during snow-and-ice and control days.

There was a sharp increase in cases of fractures of the arm, forearm and wrist (RR 3.2 (CI 1.4 to 7.6) and 2.9 (CI 1.5 to 5.4), respectively). Similarly, there was also an increase in the number of fractures of the leg (4.9 times more common) and ankle and foot (1.9 times more common; table 1).

**Table 1:** Numbers of fractures at different sites sustained on control and snow-and-ice days and their associated RR compared to 33 years ago

Fracture of	Snow-and-ice days	Control days a year later	RR (CI)	RR 33 years ago
Arm	20	7	3.2 (1.4 to 7.6)*	3.7
Forearm and wrist	33	13	2.9 (1.5 to 5.4)*	7.3
Hand	14	10	1.6 (0.7 to 3.5)	0.94
Spine and chest	2	4	0.6 (0.1 to 3.1)	1.75
Hip	11	10	1.2 (0.5 to 2.9)	3.5
Leg	13	3	4.9 (1.4 to 17)*	3.7
Ankle and foot	27	16	1.9 (1.0 to 3.5)	2.55
Total	120^†^	63	–	–

*p<0.01.

^†^There were four facial fractures in our study that were not included in this table. This is because Ralis *et al* did not include facial fractures in his original study, and therefore we did not include those in this comparative table.

## Discussion

Understanding the impact of weather variation on ED admissions can help develop strategic planning and allocation of resources.^2^ This in turn may contribute towards safer and more efficient patient care. The ED at the University Hospital of Wales, UK, currently serves a population of 482 000 people compared to a population of ∼400 000 33 years ago.^3^ ^6^ ^7^

Thirty-three years ago, Ralis *et al* published his paper on fracture incidence in snow and ice looking at data from our University Hospital ED.^3^ Ralis found that the overall risk of sustaining a fracture during snow-and-ice conditions increased by 2.85-fold.^3^ Seven years later, Ralis *et al* studied fracture incidence in snow-and-ice conditions in six UK towns. His study revealed an average of 4.5-fold increase in risk of fracture.^4^ He concluded that the UK is not adequately prepared to deal with sudden, yet predictable increase in fractures associated with snow and ice.^4^

By faithfully replicating Ralis’ methodology, accepting its limitations, we had the unique opportunity to compare the impact of snow and ice in a population of a similar magnitude attending the same ED 33 years later. We acknowledge that this paper is not a comprehensive epidemiological analysis of how changes in the weather influences fracture rates, but it does aim to give some interesting insights of how things may have changed over the past three to four decades. Were we not approaching this study bound by faithful replication of the original study methodology, with a need to compare and contrast historical data with current data, a more robust epidemiological method would have been applied. This would have included a longitudinal, prospective, population-based cohort study of the people of Cardiff, where a large population is defined and followed up prospectively for a number of years. Relative risks, along with CIs, could then have been calculated to estimate the contribution of snow-and-ice conditions to the frequency of fracture due to slipping and those due to other mechanisms. Subsequent stratification by age, sex, anatomical site of injury and mechanism could also have been performed.^8^

Similar to 33 years ago, the overall number of patients presenting with a fracture during snow-and-ice conditions more than doubled compared to control conditions. The overall risk remains similar today with a 2.20-fold increase (CI 1.7 to 3.0, p<0.01), compared with 2.85 in Ralis’ study.

Other studies have also highlighted that there is an increase in incidence of fractures and soft tissue injuries during snow-and-ice conditions.^1^ ^2^ ^5^

When stratified for age, the overall increase in fracture risk during snow-and-ice conditions is most apparent in the 31–60 years age group, a similar increase in magnitude to 33 years ago. However, in the elderly population (over 61 years age group), the increased risk of fractures dropped by 1/3 of that of 33 years ago. In addition, there was no increase in fractures associated with snow and ice in the young (0–15 years), indicating a significant improvement to 33 years ago.

The male/female distribution of fractures is similar to 33 years ago, with the over 61 years demonstrating preponderance of women sustaining fractures and the 60 years and under being roughly equal.

With regard to anatomical distribution, and again within the constraints of the methodology outlined by Ralis, the relative risk of leg and hand fractures increases with periods of snow and ice and is of a similar magnitude or even marginally higher to 33 years ago. In addition, an increase in risk of fracture to the forearm and wrist remains associated with snow-and-ice days, although the relative risk is half than that of 33 years ago.

Interestingly, there was no increase in hip fractures during snow-and-ice periods, a significant improvement to 33 years ago, where the relative risk of sustaining a hip fracture was as high as 3.5. Furthermore, no increase was seen in spine and chest fractures associated with snow-and-ice periods, compared with a relative risk of 1.75 33 years ago.

The importance of prevention strategies such as the gritting and salting of roads and footpaths as well as cleaning slippery pavements has been recommended in reducing fracture incidence during snow-and-ice conditions.^3^ ^4^ The overall improvement in fracture risk associated may be a reflection on some improvements in this area. However, while there are significant improvements in some demographic and anatomical groups, others remain unchanged.

Broadly speaking, we propose that factors contributing towards fracture risk in snow-and-ice conditions seen can be attributed to medical as well as non-medical preventative strategies that may influence the groups differently. For example, non-medical preventative strategies such as school closure and improvement in playground safety effects the young, as does possible behavioural change in the over 61 years old, who may be discouraged to venture outside by media broadcasts. It could be postulated that the group with least improvement in fracture risk who are at a working age are likely to continue with outdoor activities and therefore subject to the risks associated with snow-and-ice conditions. A similar finding was noted in a recent study in the Netherlands.^5^

In addition, and in keeping with conclusions in a paper by Murray *et al*^2^ who looked at fracture epidemics during severe weather warnings, the anatomical distribution of fractures in our study also suggested that the increase was mostly seen in the ‘walking wounded’. Murray's paper and our results suggest that the incidence of injuries such as hip and vertebral fractures, in which hospital admission is probably required, is not increased in extreme weather conditions.

From a medical preventative viewpoint, the past decades have witnessed the development and administration of therapeutic treatments, such as bisphosphonates and other agents for osteoporosis. These have been shown in randomised controlled trials to decrease the risk of vertebral and in some instances non-vertebral fragility fractures in elderly populations.^9^ ^10^ While a direct relationship between the introduction of such agents and the reduction in vertebral and hip fracture relative risk during snow-and-ice conditions cannot be made, the reduction compared to the pre-bisphosphonate era 33 years ago is noted.

A review of the literature suggests that countries with colder climates report a less dramatic increase in incidence of fractures during snow-and-ice conditions.^1^ In countries less used to long periods of snow and ice, such as UK or the Netherlands, the incidence can be doubled or even tripled.^4^ ^5^ Therefore, the fracture burden associated with snow and ice may cause a capacity–demand mismatch. Understanding this relationship and learning from the countries with colder climates could help inform the planning of a more efficient service and improve prevention strategies.

## Conclusion

Understanding the seasonal impact of weather variation on fracture incidence can help develop strategies and allocation of appropriate resources. Although the past 33 years have witnessed some improvement in fracture frequency presenting to our ED, a significant increase remains during snow-and-ice conditions. In summary, the overall number of patients presenting with a fractures during snow-and-ice conditions is still more than double compared to control conditions. Although some improvements appear to have been made during the past 33 years, this paper highlights the continued increase in frequency of some fractures and underpins the need for improving the effectiveness of preventative measures.
